# Changing concepts in perihilar cholangiocarcinoma resections and surgery

**DOI:** 10.1097/JS9.0000000000005091

**Published:** 2026-04-16

**Authors:** Eduardo Fernandes, Shintaro Yagi, Kaichiro Kato, Orlando Torres, Mario De Bellis, Hugo Pinto Marques, Andrea Ruzzenente

**Affiliations:** a Department of Surgery and Transplant, DHR Health, McAllen, Texas, USA; b Department of Hepatopancreatobiliary and Liver Transplant Surgery, Maranhão Federal University, São Luís, Brazil; c Department of Hepato-Biliary-Pancreatic Surgery and Transplantation/Pediatric Surgery, Kanazawa University, Kanazawa, Japan; d Department of Surgery, Dentistry, Pediatrics and Gynaecology, Division of General and Hepatobiliary Surgery, University of Verona, G.B. Rossi University Hospital, Verona, Italy; e Hepato-Biliary-Pancreatic and Transplantation Centre, Curry Cabral Hospital/Local Health Unit of São José, and NOVA Medical School, Lisbon, Portugal

**Keywords:** adjuvant and neoadjuvant therapies, minimally invasive surgery, perihilar cholangiocarcinoma, surgical resection

## Abstract

Perihilar cholangiocarcinoma (PHCC) is a malignant tumor arising from bile ducts at the hilar area. It was first described by Altemeyer *et al* in 1957 in Cincinnati and later in 1965 by Gerald Klatskin at Yale University. The surgical management with R0 resection represents the only potentially curative treatment for PHCC patients. The ideal surgical treatment represents a challenge for liver surgeons across the globe, and expertise in vascular reconstruction and transplantation is required. The anatomy of the liver hilum is a complex area where the proximity of portal vein bifurcation and hepatic artery branches, especially the right hepatic artery, gets an intimal relation with the bile duct confluence area. This explains frequent vascular involvement in such tumors. The modern surgical approaches in PHCC involve several topics of discussion and controversies among hepatobiliary surgeons. Some key points will be discussed in this comprehensive discussion.

## Introduction

Perihilar cholangiocarcinoma (PHCC) is a primary biliary cancer arising at the confluence of the right and left hepatic ducts. The complexity of its anatomical location – often involving the biliary tract, vascular structures, and liver parenchyma – presents a formidable challenge for resection, which is currently the only potentially curative treatment. This review provides a comprehensive analysis of the evolving concepts in PHCC management, emphasizing changes in resection techniques, diagnostic strategies, and the potential role of adjuvant and neoadjuvant therapies.

## Surgical strategies and ongoing challenges

**Figure 1.**
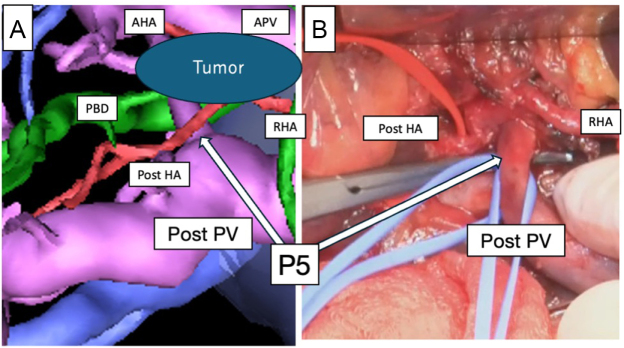
Simulation of a left tri-sectionectomy for perihilar cholangiocarcinoma (Bismuth Type IV). In left tri-segmentectomy, it is necessary to transect the portal branch toward Segment 5 (P5). However, branch of the posterior section must not be transected, as this would reduce the residual liver volume of the posterior section. Preoperative simulation using all-in-one imaging of the portal vein, hepatic artery, and bile duct allows for clear identification of P5 branching from the posterior sectoral portal vein, thereby enhancing both the safety and accuracy of the surgery. Green: bile duct, purple: portal vein, and red: artery. P5, portal branch toward Segment 5; Post PV, branch of the posterior section; APV, anterior branch of the portal vein; RHA, right hepatic artery; AHA, anterior branch of hepatic artery; Post HA, posterior branch of hepatic artery; PBD, posterior branch of bile duct.

Since Gerald Klatskin^[^1^]^ first identified these tumors in 1965, the management of PHCC has centered around achieving clear margins (R0 resection). Over time, extended resections have become the standard, and R0 resection with lymphadenectomy and vascular reconstruction is now feasible due to advancements in surgical techniques. Bismuth–Corlette classification^[^2^]^ has been widely used, which indicates tumor aggressiveness. However, it describes biliary involvement and does not account for vascular invasion, which is crucial for determining resectability. Accordingly, several classification systems, Gazzaniga Staging^[^3^]^, the Memorial Sloan-Kettering Cancer Center staging system^[^4^]^, the American Joint Committee on Cancer/Union for International Cancer Control tumor-nodemetastasis system^[^5^]^, Japanese Society of Hepato-Biliary-Pancreatic Surgery Classification^[^6^]^, and Dutch Classifi-cation^[^7^]^ have been developed to evaluate the extent of tumor involvement and guide surgical decision-making. Detailed preoperative imaging assessment is essential for surgical planning, and in particular, the use of 3D imaging to visually understand the complex hilar anatomy has become increasingly recognized as a valuable tool in formulating surgical strategies^[^8^]^ (Fig. 1).

HIGHLIGHTS

- Hepatic resection and the bile duct associated with en-bloc caudate resection with R0 remains the only potentially curative approach for perihilar cholangiocarcinoma.
- Three-dimensional imaging and functional liver reserve assessments have become integral in planning safe and effective resections.
- Advanced techniques like associating liver partition and portal vein ligation for staged hepatectomy and hepatopancreatoduodenectomy have expanded the boundaries of resectability in perihilar cholangiocarcinoma.

## Advances in the evaluation of liver functional reserves

### Future liver remnant (FLR) volume

Future liver remnant (FLR) volume is calculated by computed tomography volumetry. Various software packages have been developed for this purpose. Liver resection of PHCC with extrahepatic bile duct resection is considered to require more residual liver volume than liver resection without bile duct resection. Many centers consider FLR/total liver volume < 40% as a risk factor for postoperative liver failure after hepatectomy for PHCC and an indication for portal venous embolization^[^9^]^. Recently, FLR/body weight < 0.65%^[^10^]^ is also reported as one of the risk factors for post-hepatectomy liver failure (PHLF).

### Indocyanine green (ICG) test: ICGK and ICGK-F

The indocyanine green (ICG) test is performed frequently, especially in Japan, and is considered an essential test. However, ICG tests are not accurate in jaundice and should only be performed when serum bilirubin <3 mg/dl. High volume centers dealing with cholangiocarcinoma have established various criteria based on the ICG test. Yokoyama *et al*^[^11^]^ reported the usefulness of the FLR plasma clearance rate of ICG (ICGK-F, calculated as plasma clearance rate of indocyanine green (ICGK) × proportion of the FLR) and showed that ICGK-F less than 0.05 had the strongest impact on the incidence of postoperative mortality after liver resection after biliary cancer. Accordingly, ICGK-F > 0.05 is generally recommended for hepatic resection of PHCC as the function of the remaining liver.

## Evolving surgical techniques for extended resection

### R0 resection and radial margins

Definition criteria for R0 resection in PHCC are not entirely uniform^[^12^]^; however, in most centers worldwide, R0 resection is defined as the absence of tumor cells at the surgical margin under microscopic examination^[^13–15^]^. Seyama *et al* have proposed that a margin distance of at least 5 mm may be desirable for a more favorable prognosis^[^16^]^, this is not universally adopted as the definition of R0.

Survival rates for patients with PHCC are better in patients without lymph node metastasis, a well-differentiated tumor, and with a negative surgical margin. Of these prognostic factors, the resection margin is the only one that could be modified by the surgeon, affecting long-term survival. The other two are tumor-related factors determined at presentation. Obtaining clear resection margins has always been the core issue of surgery, as a negative margin can increase the chances of long-term survival. The R0 resection reported in the literature ranges from 19% to 95%^[^17^]^. Several factors contribute to the differences observed between studies, including patient selection, surgical strategies, complexity of surgical specimens, and the radial margin (RM) status^[^13,14,18^]^.

**Figure 2.**
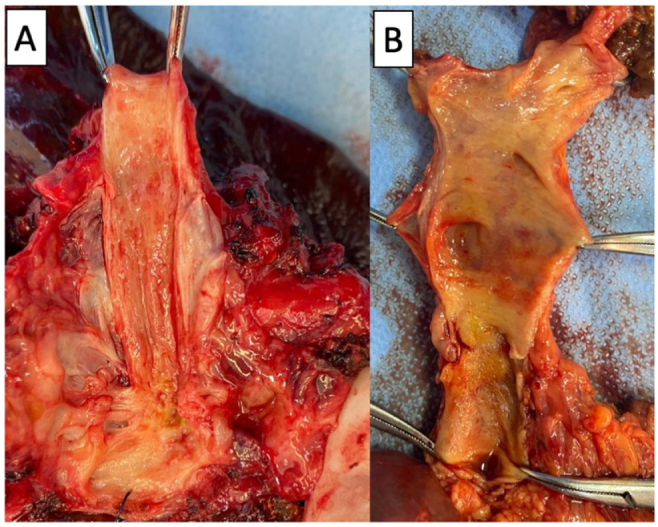
(A) Ductal margin of the distal bile duct. (B) Ductal margin of the intrahepatic bile duct.

The understanding of the surgical concept of the margin of the resected specimen in PHCC is a determinant factor in defining the extent of the disease and is divided into radial (circumferential) and ductal (longitudinal). The ductal margin (DM) represents the proximal (intrahepatic bile duct stump) and distal (common bile duct stump) (Fig. 2). The RM is classified as periductal (dissection plane of periductal tissue in the hepatoduodenal ligament), parenchymal (transection of the liver parenchyma), and vascular (portal vein and hepatic artery)^[^13,14,18,19^]^.

Proper pathological examination of residual disease should consider both DM and RM status. The evaluation of RM status is associated with survival outcomes. This concept highlights that PHCC requires extensive peritumoral resection, including vascular resection and reconstructions associated with liver resection. The definition of R1 resection for PHCC was recommended by the Royal College of Pathologists and the International Collaboration on Cancer Reporting as the same used for pancreatic ductal adenocarcinoma. Therefore, microscopically residual disease was considered if the cancer cells were detected in less than 1 mm from the transection or dissection margin^[^13,14,18^]^.

### Lymph node dissection

Lymph node metastasis is common in PHCC, the anatomy surrounding the hilar bile duct is complex, and the extent of dissection in radical resection for PHCC is still under debate. Lymphadenectomy is a crucial part of the modern approach of radical resection for PHCC and is one of the most relevant factors affecting prognosis. The hepatopancreatobiliary (HPB) surgeon should be aware of the lymphatic drainage pathway of the hilar bile duct to guide the ideal lymph node dissection for PHCC. Detailed anatomical knowledge of lymph node stations, as elegantly described by Japanese researchers, provides the basis for systematic lymph node dissection in PHCC. The lymphatic metastasis pathway of PHCC is closely related to the lymphatic drainage of the hilar bile duct. Japanese researchers conducted in-depth studies of the lymphatic drainage pathway of the hilar bile duct. They discovered that the lymphatic drainage of the hilar bile duct follows three paths: (1) That from the hepatic artery (No. 12a) along the common hepatic artery (CHA) (No. 8) to the celiac lymph nodes (No. 9); (2) Along the bile duct (No. 12b) and on the posterior surface of the pancreas head (No. 13), and then to the para-aortic lymph node (No. 16); and (3) Along the portal vein (No. 12p) to reach the superior mesenteric vein and then the superior mesenteric nodes (No. 14)^[^17,20,21^]^.

Based on the previous anatomic definition of lymphatic pathways, except for the hepatoduodenal ligament lymph nodes, the lymph nodes along the CHA are the nearest station in patients with PHCC, followed by the posterior pancreaticoduodenal lymph nodes and para-aortic lymph nodes. Consistent with the previous descriptions of the lymphatic pathways, metastasis from the posterior pancreaticoduodenal lymph nodes was associated with poor prognosis in patients with PHCC compared to that in the lymph nodes along the CHA^[^17,21,22^]^.

The Japan Society of Hepatobiliary and Pancreatic Surgery (JSHBPS) defined N1 as regional lymph node metastasis that includes the cystic duct, common bile duct, hepatic artery, portal vein, and behind the pancreatic head. Distant metastasis (M1) was considered beyond these lymph nodes. According to JSHBPS, lymph nodes from groups 8, 12, and 13 should be dissected, and the para-aortic lymph node (No. 16) should be sampled routinely^[^15,17,22^]^. According to Aoba *et al*, lymph node metastasis is directly correlated with the degree of infiltration (T) and Bismuth subtype, which was 21.1% for Bismuth I, 27.3% for Bismuth II, 41.5% for Bismuth III, and 55.6% for Bismuth IV, respectively^[^15,20,22^]^.

Total lymph node count (TLNC) and lymph node ratio, although relevant for gastrointestinal tumors, are still controversial for PHCC. Moreover, accurate lymph node staging can be performed only when TLNC is obtained, as some lymph node metastases may be misdiagnosed when the total number is insufficient, leading to inaccurate tumor staging. However, the number of lymph node metastases is associated with survival. Bagante *et al* found that in patients with ≤3 lymph node metastases, the 5-year survival of patients was significantly better than that of patients with >3 lymph node metastases^[^15,23^]^.

### Caudate resection

**Figure 3.**
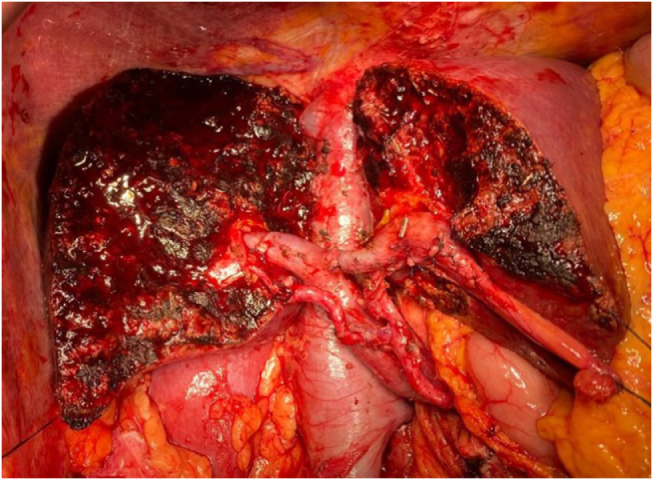
Central hepatectomy with caudate resection.

Hepatic resection and the bile duct associated with en-bloc caudate resection have been suggested for patients with PHCC (Fig. 3). The explanation for the combined liver resection, including the caudate lobe, originates from anatomical and histological studies. The anatomy of the caudate lobe’s biliary branches drains directly into the biliary confluence bilaterally to the right and left hepatic duct, which increases the risk of involvement of the caudate lobe by the tumor. The importance of caudate liver resection was shown in 1990 by Nimura *et al*. After that, the rate of caudate resection increased worldwide^[^24,25^]^.

There is strong evidence that the radicality of surgery, with routine resection of the caudate, is associated with long-term survival benefits. However, the concerns about the FLR, the hepatic reserve, perioperative morbidity, and risk of mortality balance with the clear advantage of resection of the caudate lobe. Furthermore, the extension of the caudate lobe behind the vena cava and anatomic variations could make the dissection more difficult, resulting in lower rates of caudate resection^[^24,26^]^.

Birgin *et al* in a systematic review, observed a lack of controlled clinical trials. However, the authors identified a significantly lower positive margin in patients with resected caudate lobes when they analyzed only the meta-analysis extracted from these studies. Furthermore, the caudate lobe resection was associated with improved overall survival (OS) without increased morbidity or mortality^[^26,27^]^.

### Right or left liver resection

**Figure 4.**
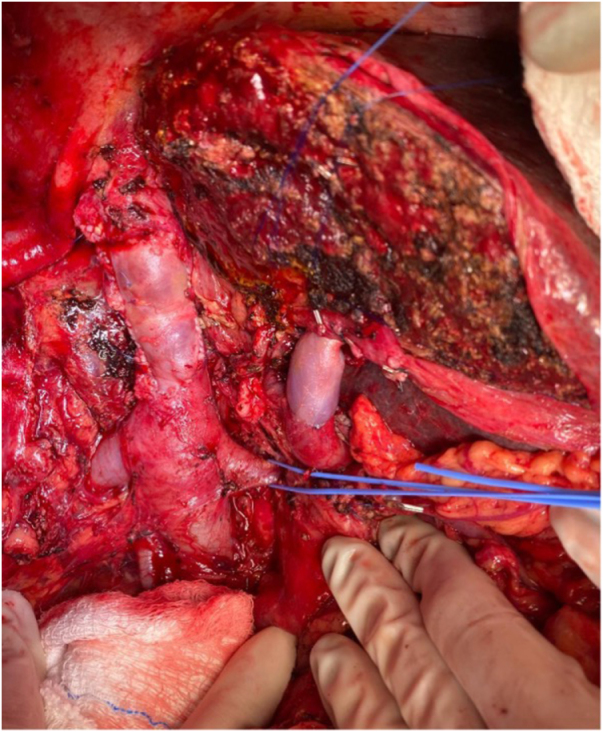
Right trisectionectomy with inferior vena cava and portal vein resection.

**Figure 5.**
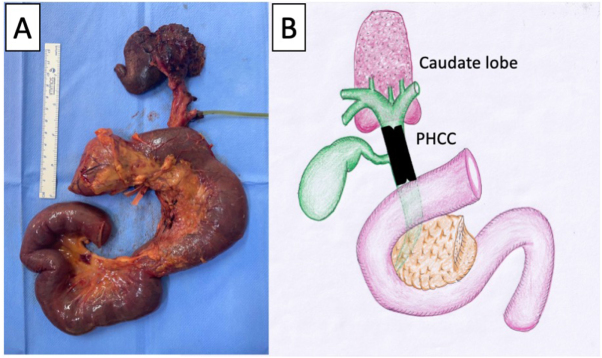
Hepatopancreatoduodenectomy with caudate resection for perihilar cholangiocarcinoma (Bismuth Type I). Resected specimen (A) and its schema (B).

Radical resection of PHCC includes a major hepatectomy combined with resection of the extrahepatic bile duct (Figs 4 and 5). In some patients, unilateral vascular involvement and liver atrophy suggest the side of liver resection. However, right or left-sided liver resection can achieve similar complete (R0) resection, 5-year survival, and portal vein reconstruction rates. In general, the predominance of the side of the involvement defines the type of resection: right liver resection for type IIIa and IV with predominant right-side tumor involvement and left liver resection for type IIIb and IV with predominant left-side tumor involvement. In centrally located tumors, HPB surgeons prefer right liver resection due to some anatomical considerations. The longer the left extrahepatic DM, the right-sided lie of the bile duct confluence, the right hepatic artery located behind the common duct, commonly involved by the tumor, and anatomical variations being more frequent on the right side, are advantages for right-sided hepatectomy. However, the oncological superiority of right-sided hepatectomy over left-sided hepatectomy is not supported. Furthermore, a smaller FLR in right-sided hepatectomy, when compared with left-sided liver resection, leads to a higher risk of postoperative liver failure associated with higher postoperative mortality. Left liver resection is associated with a significantly higher requirement for right hepatic artery reconstruction^[^27–29^]^.

The relationship of the biliary tumor at the hilum with the hepatic inflow often defines the extent of liver resection. It is best assessed by cross-sectional imaging performed before biliary decompression. The right hepatic artery commonly courses behind the common bile duct. If the right hepatic artery is involved by the tumor located on the left side, the resection requires right hepatic artery reconstruction, or it is considered unresectable^[^28–30^]^.

To determine the ideal surgical procedure for PHCC of left-side predominance, the relationship between the most peripheral infiltrated right posterior sectional bile duct (RPSBD) and right portal vein is crucial because the anatomy of the RPSBD resection line in left hepatectomy is restricted at the craniodorsal border of the right portal vein (supraportal type). Based on this, when peripheral biliary invasion is localized within the craniodorsal border, left hepatectomy may be generally indicated for PHCC of left-side predominance. However, left trisectionectomy should be the procedure of choice in patients when biliary invasion extends beyond the border of the right portal vein and further to the intrahepatic bile duct. The most common reason for inaccuracy is the underestimation of the extent of the tumor^[^28–30^]^.

### The role of associating liver partition and portal vein ligation for staged hepatectomy (ALPPS) procedure

Surgical resection for PHCC requires an aggressive procedure, including extended liver resection, vascular resection, and in some cases, pancreatic resection to achieve R0 resection and consequently long-term survival. However, there is a high risk of insufficient FLR and PHLF. The optimal way to avoid or minimize the risk of PHLF is still under debate. The FLR volume and total liver volume ratio are vital for surgical planning, particularly in a damaged liver due to chemotherapy and/or cholestasis. Moreover, the comorbidities, the extent of the resection, performance status, and biliary and vascular invasion represent a challenge for hepatobiliary surgeons. For a safe liver resection, parenchymal augmentation techniques are necessary for surgical planning, such as portal vein embolization, liver venous deprivation, and ALPPS. ALPPS technique has been introduced in clinical practice as the biggest and fastest hypertrophy compared to other modalities to achieve augmentation. ALPPS can shorten the length of the interstage period, minimizing the risk of tumor progression, reducing dropouts due to complications, and increasing the resectability rates^[^29–31^]^.

The ALPPS technique for cholangiocarcinoma has shown a decrease in mortality. Balci *et al*, in the largest international multicenter cohort, including 39 patients who underwent ALPPS for cholangiocarcinoma, observed a 90-day mortality of 7.7% (3/39 patients) in experienced centers^[^32^]^. All cases completed two-stage right-sided resections with right trisectionectomy in most cases, with an >80% R0 resection rate without significant infection/ cholangitis in most patients before stage 2. Moreover, the median number of resected lymph nodes was 5.5, in line with the reported benchmark range of ≥4 in classical PHC surgery^[^31,33,34^]^.

Bile leak is an important negative factor in survival after ALPPS for cholangiocarcinoma. Therefore, meticulous parenchymal transection at stage 1 and biliary anastomosis could potentially reduce this complication. In addition, biliary anastomosis should be performed at stage 2 to decrease the risk of bile leak. Another negative factor is the routine concomitant vascular resection in most patients. Balci *et al* observed that patients who had vascular resection during stage 2 experienced early mortality, suggesting that when resection is necessary, the procedure should be performed in stage 1 with a clear and untouched operating field^[^32^]^.

The early mortality for hepatocellular carcinoma that underwent ALPPS ranges from 11 to 31%, and for liver metastases of colorectal cancer ranges from 4.9 to 9.1%. For PHCC, a 7.7% early mortality rate is perfectly acceptable in this setting when compared to the 13% benchmark cutoff in low-risk PHCC patients. The learning curve has an effect on ALPPS for PHCC as the early mortality occurs in procedures performed in the first half of the study period^[^29–31,33,34^]^.

The ALPPS procedure is not associated with higher mortality rates than other major surgical procedures and does not cause potentially less oncological efficacy. Adequate patient selection and centers with expertise in complex hepatobiliary surgery are important to achieve R0 resection with lower mortality rates^[^30,31^]^.

### Hepatopancreatoduodenectomy (HPD)

**Figure 6.**
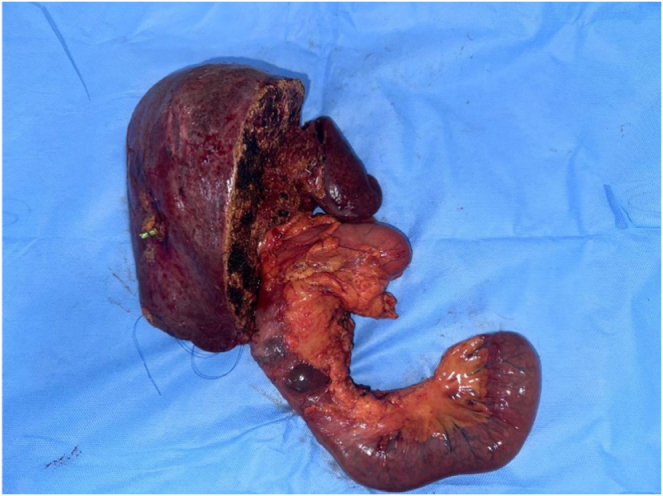
Hepatopancreatoduodenectomy including right hepatectomy and caudate resection.

**Figure 7.**
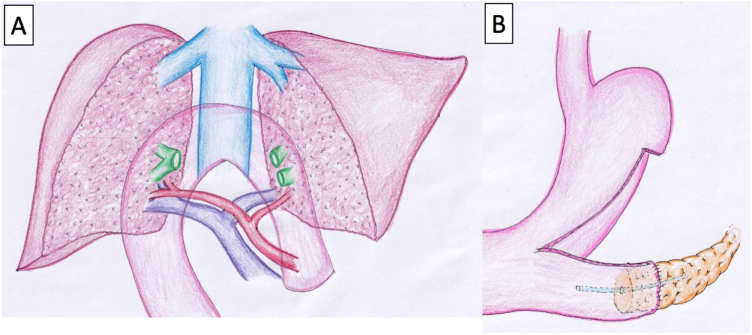
The schema of bilateral biliary reconstruction (A) and pancreaticogastrostomy (B) after hepatopancreatoduodenectomy.

Hepatopancreatoduodenectomy (HPD) is the combination of liver resection, pancreaticoduodenectomy, and resection of the entire extrahepatic biliary system simultaneously (Figs 5-7). HPD has been used for the treatment of selected patients with PHCC invading both the intrapancreatic common bile duct and the hepatic hilum. These patients have been historically considered to have unresectable tumors. This demanding extended multivisceral resection has been a great challenge to HPB surgeons. HPD is a major surgical procedure and is associated with high morbidity and mortality rates and controversial survival benefits^[^35,36^]^.

In 1986, the first HPD, including the hepatoduodenal ligament, was performed in a patient with advanced gallbladder cancer. HPD for biliary cancer was performed thereafter. All surgeries should be performed after the serum total bilirubin levels are less than 3 mg/dl. The surgery is indicated after multidisciplinary discussion in an extensive spread of cholangiocarcinoma that otherwise could not be removed completely and not achieving R0 resection. Pancreatoduodenectomy is commonly the first procedure, followed by a complete upward lymphadenectomy at the hepatoduodenal ligament. The dissection of regional lymph nodes, including the pericholedochal, periportal, and CHA nodes, is routine. After that, the ipsilateral hemiliver, caudate lobe, and liver are mobilized along the hepatic vein, followed by division of the intrahepatic bile duct. Combined vascular resection is necessary in some cases during the dissection of the hepatoduodenal ligament, including portal vein resection and hepatic artery resection^[^36–38^]^.

The overall prognosis of HPD for biliary and gallbladder cancers remains dismal, particularly related to the advanced stage of the disease at presentation. In specialized centers, the procedure has been performed to improve the resectability and achieve R0 resection. However, this aggressive technique remains controversial, and every case should be sent to the multidisciplinary discussion as postoperative morbidity occurred in almost 80% of patients and the mortality is around 10%. PHLF is the most reported complication due to insufficient FLRs. To improve the safety and tolerance of major liver resections, preoperative portal vein embolization has been proposed^[^38,39^]^.

Another source of PHLF is preoperative hyperbilirubinemia, as biliary obstruction increases susceptibility to endotoxemia and impairs the function of hepatocyte mitochondria. Preoperative biliary drainage is mandatory to achieve a bilirubin level of less than 3 mg/dl, promoting early bile duct decompression in the FLR, preventing cholangitis, and improving liver function^[^36,38,39^]^.

## Minimally invasive approaches

Minimally invasive surgery (MIS) has experienced a notable increase in utilization and is progressively refining the management of PHCC, despite initial concerns about technical difficulties and extended learning curves^[^40^]^. It should be noted that MIS approaches for PHCC are at early stages of development, so patient selection could be biased. Taking this limitation into account, MIS offers comparable outcomes to open surgery in terms of 90-day mortality, number of lymph nodes retrieved, R0 resection rates and estimated survival rates^[^41^]^. MIS is more time-consuming and technically challenging but has the added benefits of less blood loss, shorter hospital stays, and reduced morbidity. Moreover, the faster recovery and decreased postoperative complications observed after MIS could increase the likelihood of receiving adjuvant chemotherapy^[^42^]^. Conversion rate in MIS is low and mainly related to intraoperative findings as the unclear tumor extent rather than unexpected intraoperative adverse events^[^43^]^. Hence, MIS appears to be both feasible and safe for PHCC^[^44^]^.

Though a proper comparison between laparoscopic and robot-assisted surgery in PHCC is missing, the robotic approach may provide additional advantages over the laparoscopic approach in those cases requiring complex hilar dissection and biliary reconstruction. Robotic platforms provide outstanding magnification and 3D visualization of the surgical field. Furthermore, the improved dexterity of “wristed” robotic tools with additional degrees of freedom may facilitate fine dissection and suturing as required for lymphadenectomy, caudate lobectomy, multi-duct deep-seated hepatico-jejunostomy and vascular resection^[^45^]^.

Data on MIS in PHCC are currently scarce and come from retrospective studies that include highly selected patients, so they are insufficient to demonstrate the superiority of laparoscopic or robotic techniques over open surgery^[^45^]^. MIS for PHCC should be carried out in high-volume hepatobiliary units by skilled minimally invasive surgeons to guarantee safe postoperative outcomes, adequate oncology radicality, and proper management of unforeseen complications.

## Adjuvant and neoadjuvant therapies

Surgical resection with a histologically negative margin is the only potentially curative treatment for PHCC^[^46^]^. Nevertheless, most patients are not suitable for surgery as they present with locally advanced disease or distant metastasis^[^47^]^. Even after curative resection, recurrences develop in more than half of cases resulting in poor survival^[^48^]^. This necessitates consideration of adjuvant and neoadjuvant treatments.

### Adjuvant chemo- and radio-therapy

Limited data exist regarding the impact of adjuvant therapy on PHCC. The French PRODIGE-12/ACCORD-18^[^49^]^ and Japanese BCAT^[^50^]^ Phase III studies did not show any significant enhancement in relapse-free survival (RFS) or OS with adjuvant therapy. Instead, the UK BILCAP phase III trial^[^51^]^, which randomly assigned patients to receive adjuvant capecitabine or placebo, reported an increase in OS in the per-protocol analysis, although not in the intention-to-treat analysis. The BILCAP trial is currently the largest study dedicated to biliary tract cancer (BTC) and also enrolled a substantial number of PHCC patients. The study design involved 1:1 randomization with stratification of main prognostic factors like primary tumor location and resection margins. Even though the BILCAP study did not categorize based on lymph node metastases, such characteristics were well balanced between study arms, and it is unlikely that this influenced the results. In contrast to other phase III trials, the BILCAP study required patients undergoing surgery at specialized hepato-pancreato-biliary centers, a standardized definition of R1 and showed a longer follow-up time. One key critique of the BILCAP trial is the modest decrease in relapse rate (65 vs. 60%) and the lack of long-term benefit in RFS after 24 months, suggesting capecitabine may only delay recurrence^[^52^]^. Despite these reservations, the BILCAP trial has shifted the paradigm of adjuvant therapy, establishing capecitabine as the new standard of care for resected BTC.

Unfortunately, the rate of relapse is still high, indicating that not all patients have positive results from this additional treatment. This highlights the need for further randomized research to investigate the role of new strategies^[^53^]^. Based on the BILCAP trial results, NCCN (National Comprehensive Cancer Network), ASCO (American Society of Clinical Oncology), and ESMO (European Society for Medical Oncology) guidelines recommended adjuvant capecitabine^[^54–56^]^.

The role of adjuvant radiotherapy (RTx) or chemo-radiotherapy (CRTx) remains controversial, with no prospective randomized controlled studies on this issue. While some retrospective studies focusing on PHCC commonly supported the necessity of adjuvant RTx^[^57^]^ or CRTx^[^58^]^, there is a lack of consensus regarding patient selection. Some observational studies found significantly improved OS after CRTx in R1^[^59^]^ or N1 resection^[^60^]^, which are the main factors negatively affecting survival in PHCC patients. However, the added value of RTx needs to be further investigated in a study with a higher level of evidence.

### Neoadjuvant chemotherapy

Currently, there is no standard of care or prospective data suggesting the administration of neoadjuvant chemotherapy (NAC) in the treatment of resectable or advanced PHCC^[^61^]^. Regrettably, many survival studies group various BTC subtypes together, although they present different biological behavior, due to the rarity of the tumors^[^61,62^]^. Up to date very few PHCC patients are candidate to NAC, however its use has risen in recent years^[^62^]^. Several retrospective observational studies suggest the potential management strategy of a pre-operative treatment. In theory, the NAC strategy could potentially offer many benefits. First, the downstaging of the tumor to improve resectability and odds of resection with negative margins, second, the treatment of occult systemic disease that cannot be detected by preoperative imaging^[^63^]^. There are experiences reporting NAC enables the downstaging/downsizing of borderline resectable patients and conversion surgery in selected locally advanced patients leading to better prognosis^[^64^]^. Moreover, NAC assists with patient selection by identifying those patients with aggressive PHCC who quickly develop overt metastatic disease and avoiding futile surgery. Lastly, NAC use is also promoted by the fact that the high rate of postoperative complication and longer recovery period after PHCC surgery often prevent the administration of adjuvant therapy^[^65^]^. The administration of NAC seems to be feasible and well-tolerated without affecting liver function and postoperative course^[^66^]^. Data from ongoing prospective clinical trials may clarify the effectiveness of NAC relative to upfront surgery^[^67^]^.

## Conclusion

The management of PHCC has evolved substantially in recent years, with improved diagnostic accuracy, advanced surgical techniques, and emerging systemic therapies offering new hope for patients. While the standard of care remains R0 resection with lymphadenectomy, evolving surgical concepts in MIS, and NAC/adjuvant therapy hold promise for improved survival. Continued research and a multidisciplinary approach are essential to further refine PHCC treatment and improve outcomes.

## Data Availability

Because this manuscript is a review paper, we did not use clinical data except for the image of a case.
